# Prevalence and Clinical Manifestation of Astrovirus Gastroenteritis in Adults: A Seven-Year Study in Washington D.C., USA

**DOI:** 10.3390/v17050730

**Published:** 2025-05-20

**Authors:** Maryam Mehdipour Dalivand, Maher Ali, Rebecca Yee

**Affiliations:** Department of Pathology, George Washington University School of Medicine and Health Sciences, Washington, DC 20037, USA

**Keywords:** astrovirus, gastroenteritis, syndromic testing, gastrointestinal PCR

## Abstract

Gastroenteritis is commonly caused by viral etiologies. The inclusion of astrovirus on multiplex, syndromic gastrointestinal PCR panels allows for the detection and characterization of infected patients. This retrospective, observational, clinical study examines the epidemiology and clinical characteristics of astrovirus infections in adults from our institution in Washington D.C. (USA) over a seven-year period. Chart abstraction was performed to collect patient demographics, laboratory results, clinical presentation, and management. The overall positivity rate of astrovirus was 0.6%. Peak seasons were late winter to spring (February–April). The mean age was 32 years old (range: 18–52 years). All patients presented with gastroenteritis symptoms and were immunocompetent except one. Symptoms varied among diarrhea, abdominal pain, vomiting, and fever, but patients in age group 30–39 years experienced less vomiting (*p* = 0.01). Infected patients had an increase in monocytes and neutrophils and a decrease in lymphocytes (*p* < 0.001). Gastrointestinal co-infections were seen in 24% of our patients. In all patients, clinicians acknowledged the detection of astrovirus and discharged patients without further treatment. The median length of stay was 6 h, and no patients were admitted into the intensive care unit. We show that astrovirus infections in immunocompetent adults were associated with mild disease associated with specific cell counts and different symptoms correlated with age.

## 1. Introduction

In the past decade, the global burden of diarrheal diseases has remained a public health challenge, particularly in low-resource settings where these conditions rank among the leading causes of death [1]. Diarrheal diseases, exacerbated by poor sanitation and limited access to clean water, have a significant impact on healthcare systems worldwide. Viruses are the leading cause of acute gastroenteritis, followed by bacteria and parasites [2].

Human astroviruses, discovered in 1975, are positive-sense single-stranded RNA viruses. They are a notable cause of mild diarrhea, particularly important as a nosocomial agent capable of causing outbreaks and hospitalizations [3]. Astroviruses primarily affect children, causing mild watery diarrhea lasting 2–3 days, often accompanied by vomiting, fever, anorexia, and abdominal pain [4,5]. However, the virus also poses a risk to immunocompromised individuals and the elderly. The mean incubation period for astroviruses infections is approximately 4.5 days [6].

Currently, the medical community possesses advanced diagnostic tools and a broad understanding of common viral pathogens [7,8]. However, there is a significant gap in the understanding of less studied viruses like astroviruses, especially in adult populations. Although children are commonly affected and reported, the impact on adults is not as well documented. This discrepancy highlights a crucial need for enhanced research across all demographics.

The advent of molecular syndromic testing has enabled rapid testing for infectious etiology of gastroenteritis [9]. At our institution, we implemented the BioFire^®^ FilmArray^®^ Gastrointestinal panel (BioFire Diagnostics, LLC, Salt Lake City, UT, USA), which can simultaneously detect 22 targets consisting of bacteria, viruses, or parasites [9,10]. While viral targets such as norovirus, adenovirus, or rotavirus are common viral causes of gastroenteritis, the panel also detects astrovirus. Utilization of this panel allows clinical laboratories to readily uncover targets not commonly detected and thereby contribute significantly to the existing body of knowledge regarding their prevalence and impact. This paper presents the epidemiology and clinical characteristics of astrovirus infections in adults from our institution over a period of seven years.

## 2. Materials and Methods

### 2.1. Study Design

We performed a single-center, observational, retrospective study at The George Washington University Hospital, which is a 395-bed tertiary care, academic medical center with a level I trauma center located in Washington D.C., U.S.A. Stool specimens submitted between January 2016 to March 2023 were included. The detection of astrovirus was made by testing stool specimens from patients using the qualitative, multiplex, BioFire^®^ FilmArray^®^ Gastrointestinal Panel (GIPCR) [9,10]. Stools were tested following the manufacturer’s instructions. Results were reported as detected or not detected. At our institution, the GIPCR test is available to patients who are actively experiencing diarrhea, are not on laxatives, and with specimen collected typically within 72 h of admission (including the initial time of visit into the Emergency Department (ED) if that was the first location of admission). The laboratory will reject the order if the stool specimen submitted is formed stool and repeat testing is blocked within 7 days of initial testing.

The following patient information was abstracted from electronic medical records: (i) demographics such as age, gender, the presence of underlying medical conditions, and immune status; immune status was determined by evaluating defects in the immune system from underlying diseases (e.g., malignancies, HIV infection, malnutrition), medical treatments (e.g., chemotherapy, glucocorticoids, biologic agents) for conditions such as solid tumors or hematologic malignancies, or receipt of solid-organ or stem cell transplants with ongoing immunosuppression, moderate to severe primary immunodeficiencies (e.g., CVID, SCID, DiGeorge syndrome) and interventions (e.g., total body irradiation, splenectomy); (ii) clinical symptoms on presentation and all recorded ICD-10 (10th revision of the International Classification of Diseases) codes; (iii) laboratory results pertaining to stool testing such as Ova-and-parasite testing, stool culture, and complete blood cell counts; and (iv) clinical management (e.g., drugs or biologics administered).

### 2.2. Statistical Analysis

Data were statistically described in terms of mean, range, and relative frequencies (%) when appropriate. Comparisons between groups were done using the student’s *t*-test for normally distributed quantitative variables, and the Chi-Square Goodness-of-Fit Test was performed for observed distribution of parameters (e.g., cell blood counts). *p* < 0.05 was considered statistically significant.

## 3. Results

### 3.1. Prevalence and Seasonality of Astrovirus

**Figure 1:**
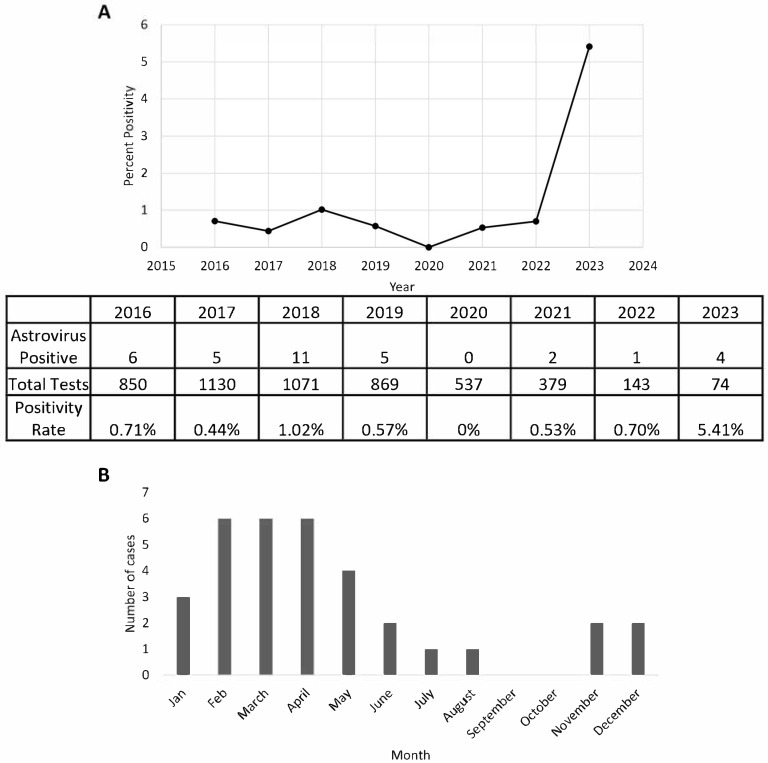
The detection of astrovirus in our patient population between 2016 and 2023. Positivity rate of astrovirus determined by using the total number of stool specimens tested on a multiplex, gastrointestinal PCR per year (**A**). Seasonality of astrovirus demonstrated by positivity per month (**B**). Data represent aggregated sum of total positives from each respective month. Data were collected from 1 January 2021 to 30 March 2023.

Between January 2016 to March 2023, a total of 5053 GIPCR tests were performed, with 34 cases of astrovirus detected, yielding an overall positivity rate of 0.6%. The highest positivity rate (1.02%) was observed in 2018 with 11 cases out of 1071 tests performed (Figure 1A). In contrast, the positivity rate in 2020 was 0%, with no positive cases detected among 537 tests performed that year. There was a notable drop in positivity after 2018, with a slight resurgence starting in 2021. Interestingly, despite fewer tests conducted in early 2023 (74 tests only), four cases of astrovirus were detected (5.4%), suggesting an increasing trend. By the time of writing this manuscript, we have stopped collecting data for the rest of 2023.

Our data further revealed that peak astrovirus incidence occurs from late winter to spring, particularly between February and April, when the highest number of cases was recorded (Figure 1B). Following this peak, incidence gradually declined, with a moderate number of cases observed in May and June. This seasonal pattern suggested that astrovirus infections are more prevalent during colder months, with a decline in warmer seasons before experiencing a minor increase toward the year’s end.

### 3.2. Clinical Characteristics of Patients with Astrovirus

The mean age of patients with astrovirus infection was 32 years (range: 18–52 years), with 44% occurring in individuals between 29 and 38 years of age (Table 1). The majority of affected patients were female, accounting for 56% of cases. In our patient population, the median length of stay in our hospital (including time spent in our ED if applicable) was 6.4 h with a range of 2.3 h–4 days. A majority of our patients were ordered a GIPCR panel from their visit in our ED (*n* = 31, 91%). Three patients were admitted from the ED into the inpatient wards for clinical reasons unrelated to gastroenteritis, such as monitoring of sepsis, pericarditis, and concussion incident. No patients were admitted to the intensive care units and no nosocomial outbreaks were identified in our hospital associated with astrovirus.

To further understand why the patients presented to the hospital and if astrovirus could be an incidental finding, we reviewed the ICD-10 (10th revision of the International Classification of Diseases) codes that clinicians included into the patient’s electronic medical records. Our findings revealed that 100% of the patients had an ICD-10 code associated with gastrointestinal infections including gastroenteritis, diarrhea, abdominal pain, nausea/vomiting, and dehydration (Table 1). The most reported symptom was diarrhea, which was present in all cases. Other frequently reported symptoms included abdominal pain (92%), vomiting (47%), and fever (35%) (Table 1). Symptom distribution analysis revealed that individuals in the age group of 30–39 years exhibited less vomiting (21% versus 65%, *p* = 0.01). Among patients for whom symptom duration could be estimated, the median length of illness was 3 days (range: 1–10 days).

### 3.3. Co-Infections and Co-Morbidities

A high rate of co-morbidities or co-infections was seen in our patient population positive for astrovirus. An overwhelming majority, 74%, of our patients presented with co-morbidities or co-infections (Table 1). In our patient population, all patients except one (positive for HIV) were immunocompetent. Co-infections with other gastrointestinal pathogens were identified in 24% (8/34) of patients with the following distribution: toxigenic *Escherichia coli* (*n* = 4), *Clostridioides difficile* (*n* = 2), *Campylobacter* (*n* = 1), Norovirus (*n* = 1), *Salmonella* (*n* = 1), and *Strongyloides* (*n* = 1). Two of our patients were infected with three gastrointestinal pathogens at the same time. Three of our patients had other gastrointestinal-related concerns such as inflammatory bowel disease, rectal cancer, and colectomy. Despite some of our patients having inflammatory bowel disease or rectal cancer, they were not on active immunosuppression or chemotherapy at the time of astrovirus positivity.

In all our patients, antibiotics were only used to treat other bacterial gastrointestinal infections. In astrovirus-positive patients, antibiotics were either stopped immediately upon result or no treatment was prescribed. The physicians also noted in their notes that astrovirus was recognized as a self-limiting pathogen and did not require antimicrobial intervention.

### 3.4. White Blood Cell Changes in Infected Patients

Given that many of our patients also had complete blood counts performed, we investigated whether there were distinct cell count patterns associated with astrovirus infections. Of all the parameters, a greater number of patients had increased monocytes, increased neutrophils, and decreased lymphocytes (*p* < 0.001), suggesting a distinct immune response pattern (Table 2). For monocytes, the range was 6–24% (absolute count mean and 95%CI: 0.7 (0.56–0.84) × 10^3^/µL), the neutrophils range was 41–86% (absolute count mean and 95% CI: 4.65 (3.88–5.42) × 10^3^/µL), and the lymphocytes range was 5–53% (absolute count mean and 95% CI: 1.29 (1.03–1.55) × 10^3^/µL). No other statistically significant changes in other parameters were observed.

## 4. Discussion

Over the study period from 2016 to 2023, an overall 0.6% positivity rate (34/5053) for astrovirus was observed in stool specimens tested. The most common presenting symptom in our patient group was diarrhea followed by abdominal pain, vomiting, and fever. Our patients ranged in age from 18 to 52 years old. Interestingly, individuals aged 30–39 years appeared less likely to exhibit vomiting. The average length of symptoms was three days, indicating a self-limited illness. We also noted that our patients harbored increased monocytes and decreased lymphocytes during time of positivity. A seasonal trend, with peak infections occurring in late winter to early spring, suggests a particular period for when clinicians should be more alert. While most studies document astrovirus infections in pediatric populations, we investigated the prevalence and trends of astrovirus in mainly immunocompetent adults in Washington D.C., USA, adding insightful epidemiology findings to this geographical area. Other studies have described the prevalence of other infectious agents causing gastroenteritis in our geographical area but omitted in-depth analysis of astrovirus due to its low positivity [8,11]. To our knowledge, this is the first study encompassing seven years of data in our patient population and such aggregated findings serve as a major strength to enhance analytical robustness.

A systematic review and meta-analysis with over 300,000 individuals revealed that the overall prevalence of human astrovirus infections was 3.48%, with the highest prevalence in children [12]. A recent study done in adult individuals, although these were oncology patients, reported a positivity rate of 0.35%, which was approximately half to our positivity rate of 0.6% [13]. We documented the highest positivity rate (1.02%) in 2018 but no positive cases in 2020. This sharp decline may reflect changes in viral circulation, influenced by pandemic-related factors such as enhanced hygiene measures or could be due to the lack of testing, all of which are possibilities driven by the stress of the COVID-19 pandemic. However, Su et al. showed in their study that viral gastroenteritis cases actually increased post COVID-19, with increasing rates shifting from children to adults [14]. Recent viral genetic studies revealed rare and recombinant genotypes of astrovirus detected in humans in rural West Africa [15]. These are all important developments suggesting that studies into astrovirus in adults are becoming more of clinical importance.

Our findings highlight the underrecognized impact of astrovirus in adult populations, demonstrating that even immunocompetent individuals can experience significant symptoms [16,17]. Understanding the prevalence in adults is important especially to reduce infections in community and healthcare settings [18]. Our findings demonstrated there was monocyte elevation and lymphocyte reduction, which may be used as diagnostic parameters. Understanding the cell counts may help determine the pre-test probability for acute viral gastrointestinal infections, such as astrovirus, so that more judicious ordering of syndromic panels like GIPCR, which has poor reimbursement rates, can be implemented. Others have shown that red blood cell distribution width can be a marker for severity in pediatric acute gastroenteritis and more commonly, increased white blood cell count can be an indicator for *C. difficile* infection [19,20]. Additionally, we also saw some symptomatology differences between males and females and age. Differences in immune response and clinical symptoms between the sexes in gastrointestinal infections have been reported in *Entamoeba histolytica*, *Salmonella*, or *Vibrio* infections where men had a higher proportion of invasive disease [21,22,23]. We have shown that for the symptom of fever, a greater proportion of adults between 30 and 39 experienced less fevers (40% versus 67%, *p* = 0.7) and was more commonly reported in males (47% vs. 26%, *p* = 0.3); although a >20% difference was observed, neither difference reached statistical significance, which could be due to the low sample size, warranting further studies to evaluate this hypothesis. Additionally, further studies are needed to explore the clinical significance of hematological changes, histopathological findings, genotypic differences in circulating isolates, and their correlation with disease severity, patient outcomes, and potential implications for adults.

Despite these insights, several limitations of our study should be acknowledged. First, this was a single-institution study which may restrict the generalizability of the data, as larger multi-center investigations would provide a more robust picture of astrovirus epidemiology. Stool ordering practices may vary by clinician, and astrovirus testing might not have been performed in all patients with gastroenteritis, potentially underestimating true prevalence. The true burden of astrovirus may also be masked by COVID-19, as changes in social behavior collectively minimized the spread of various pathogens [24]. In our hospital we used the BioFire^®^ FilmArray^®^ Gastrointestinal panel, which does have published limitations for the detection of astrovirus including failure to detect the novel, circulating clades in stool samples, suggesting that the true prevalence of astrovirus in our patient population may be underestimated [25]. While 34 astrovirus-positive cases offer meaningful observations, the small size can restrict subgroup comparisons. Nonetheless, this disease is most often reported in the pediatric population or immunocompromised individuals. A strength of this study is that our aggregated analysis can provide more in-depth findings than individual case reports and we, too, still found statistically significant phenotypes. The presence of other gastrointestinal pathogens can confound the clinical picture, making it difficult to attribute specific symptoms solely to astrovirus, although we now have insights into the co-infections with astrovirus.

In conclusion, we characterized individuals with astrovirus infections from a single institution across seven years. Our investigation underscores that adult astrovirus infections, although infrequent, can present with clinically meaningful symptoms, even in immunocompetent individuals, and these should not be overlooked.

## Figures and Tables

**Table 1:** Demographics and clinical breakdown of patients positive for astrovirus.

Parameter	Value
Demographics	
Total no. of patients (%)	34 (0.67)
No. of female patients (%)	19 (56)
No. of male patients (%)	15 (44)
Mean patient age (yrs)	32
Range of Patient Age (years)	18–52
Race/Ethnicity	
Caucasian	19 (56)
African American	8 (26)
Hispanic	4 (9)
Asian	1 (3)
American Indian	1 (3)
Unknown	1 (3)
Location of Test Order	
Emergency Department	31 (91)
Inpatient Wards	3 (9)
Symptoms/Clinical Manifestations	
Diarrhea	34 (100)
Abdominal pain	31 (92)
Vomiting	16 (47)
Fever	12 (35)
Median length of illness (days)	3
Range of length of illness (days)	1 to 10
Median length of stay	6.4 h
Range of length of stay	2.3 h to 4 days
ICD-10 codes	
Codes related to gastrointestinal infections	34 (100)
Gastroenteritis	21 (62)
Diarrhea	16 (47)
Abdominal pain	8 (24)
Nausea/Vomiting	7 (21)
Dehydration	4 (12)
Colitis	2 (6)
Fever	2 (6)
Esophagitis	1 (3)
Sepsis	1 (3)
Pregnancy	1 (3)
Shortness of Breath	1 (3)
COVID-19	1 (3)
Arthritis	1 (3)
Urinary tract infection	1 (3)
Concussion	1 (3)
Vasovagal syncope	1 (3)
Biliary disease	1 (3)
Hypertension	1 (3)
Polycystic ovary syndrome	1 (3)
Co-morbidities and Co-infections	
Previously healthy	9 (26)
Total no. of patients with co-morbidities or co-infections	25 (74)
Gastrointestinal co-infections	8 (24)
Dual infection (%)	6 (18)
Triple infection (%)	2 (6)
Toxigenic *Escherichia coli* (%)	4 (12)
*Clostridioides difficile* (%)	2 (6)
*Campylobacter* (%)	1 (3)
*Salmonella* (%)	1 (3)
*Strongyloides* (%)	1 (3)
Norovirus	1 (3)
HIV	1 (3)
Other gastrointestinal co-morbidities	3 (9)

Abbreviations: ICD-10: 10th revision of the International Classification of Diseases; no: number

**Table 2:** Complete blood counts from patients positive for astrovirus.

Parameter	Abnormal High (*n*)	Normal (*n*)	Abnormal Low (*n*)	*p*-Value	Confidence Interval, [Mean (95% CI)]	Reference Interval	Unit
White Blood Cell (*n* = 28)	2	25	1	0.9	6.89 (6.17–7.6)	4.8–10.8	×10^3^/µL
Red Blood Cell (*n* = 28)	2	22	4	0.05	4.9 (4.68–5.12)	4.7–6.10	×10^6^/µL
Hemoglobin (*n* = 28)	1	24	3	0.5	14.96 (14.37–15.55)	14–18	gm/dL
Hematocrit (*n* = 28)	2	23	3	0.2	43.59 (41.9–45.27)	42–52	%
Mean Corpuscular Volume (*n* = 28)	0	28	0	n/a	89.28 (87.79–90.76)	80–100	Femtoliters
Mean Corpuscular Hemoglobin (*n* = 28)	0	28	0	n/a	30.37 (29.9–30.83)	25.4–34.6	pg
Red Cell Distribution Width-Coefficient of Variation (*n* = 26)	2	26	0	0.6	12.79 (12.51–13.06)	11.5–14.5	%
Platelet (*n* = 28)	0	28	0	n/a	226.75 (204.73–248.77)	130–400	×10^3^/µL
Mean Platelet Volume (*n* = 27)	1	27	0	0.3	10.06 (9.77–10.34)	7.2–11.1	Femtoliters
Neutrophil (%) (*n* = 28)	17	10	1	<0.001	65.96 (60.59–71.34)	40–65	%
Lymphocyte (%) (*n* = 28)	1	9	18	<0.001	19.79 (15.51–24.06)	21–44	%
Monocytes (%) (*n* = 28)	14	13	1	<0.001	10.14 (8.55–11.73)	4.0–9.0	%
Eosinophils (%) (*n* = 26)	1	25	0	0.3	2 (0.53–3.47)	0–5	%
Basophils (%) (*n* = 25)	0	25	0	n/a	0.2 (0.04–0.36)	0–2	%
Neutrophil (#) (*n* = 28)	4	24	0	0.5	4.65 (3.88–5.42)	1.8–7	×10^3^/µL
Lymphocyte (#) (*n* = 28)	0	16	12	<0.001	1.29 (1.03–1.55)	1.0–4.8	×10^3^/µL
Monocytes (#) (*n* = 28)	3	24	1	0.5	0.7 (0.56–0.84)	0.2–1	×10^3^/µL
Eosinophils (#) (*n* = 26)	1	25	0	0.3	0.14 (0.04–0.24)	0–0.65	×10^3^/µL
Basophils (#) (*n* = 25)	0	25	0	n/a	0.02 (0.02–0.03)	0–0.2	×10^3^/µL

Abbreviation: n/a: non-applicable for statistical analysis given sample size of 0.

## Data Availability

To prevent confidentiality of the patients described in this manuscript, the de-identified data will be made available upon request.
